# Comparative efficacy of six programmed cell death Protein-1 inhibitors as first-line treatment for advanced non-small cell lung cancer: a multicenter retrospective cohort study

**DOI:** 10.3389/fphar.2024.1390872

**Published:** 2024-05-21

**Authors:** Siyuan Chen, Tao Li, Wenyu Yang, Ting Wang, Yuhui Qin, Zhijuan Du, Yanan Li, Pengfei Cui, Yi Hu, Zhefeng Liu

**Affiliations:** ^1^ Medical School of Chinese PLA, Beijing, China; ^2^ Department of Oncology, The First Medical Center, Chinese PLA General Hospital, Beijing, China; ^3^ School of Medicine, Nankai University, Tianjin, China; ^4^ Department of Medical Oncology, The Third Medical Center of PLA General Hospital, Beijing, China; ^5^ Department of Medical Oncology, Senior Department of Oncology, The Fifth Medical Center of PLA General Hospital, Beijing, China

**Keywords:** comparison, efficacy, non-small cell lung cancer, PD-1 inhibitors, long-term survival

## Abstract

The purpose of this study was to assess the comparative efficacy of six programmed cell death-1 inhibitors (nivolumab, pembrolizumab, sintilimab, tislelizumab, toripalimab, and camrelizumab) that have been used as first-line therapy for Chinese patients with advanced non-small cell lung cancer (NSCLC), which remains unclear. We determined the differences in efficacy by observing patient survival data, with the goal of informing future treatment options. Retrospective data analysis from June 2015 to April 2023 included 913 patients across six groups: nivolumab (123%, 13.5%), pembrolizumab (421%, 46.1%), sintilimab (239%, 26.1%), tislelizumab (64%, 7.0%), toripalimab (39%, 4.3%), and camrelizumab (27%, 3.0%). The median progression-free survival (PFS) for each group was 16.0, 16.1, 18.4, 16.9, 23.7, and 12.8 months, and the median overall survival (OS) was 33.7, 36.1, 32.5, not reached, 30.9 and 46.0 months for the nivolumab, sintilimab, pembrolizumab, tislelizumab, toripalimab, and camrelizumab groups, respectively. While differences existed in the objective response rates among groups (*p* < 0.05), there were no significant differences (all *p* > 0.05) in PFS or OS. The findings suggest comparable efficacy among these PD-1 inhibitors for NSCLC treatment, underscoring their collective suitability and aiding treatment decisions.

## 1 Introduction

Immune checkpoint inhibitors (ICIs), which primarily comprise programmed cell death-1 (PD-1) inhibitors and programmed cell death ligand 1 (PD-L1) inhibitors, have become the standard treatment option for patients with advanced non-small cell lung cancer (NSCLC) without driver gene mutations. Multiple PD-1 inhibitors have been approved by the United States Food and Drug Administration and the National Medical Products Administration of China as first-line treatment for advanced NSCLC. In real-world clinical practice in China, for example, six PD-1 inhibitors, including pembrolizumab, nivolumab, sintilimab, tislelizumab, toripalimab, and camrelizumab, are used to varying degrees as first-line treatment for advanced NSCLC. However, there are differences among these PD-1 inhibitors based on their clinical trials. In the KEYNOTE-407 trial, pembrolizumab was mainly used in non-Asian patients, while in the ORIENT-12 trial, sintilimab was mainly used in Asian patients (Sung et al., 2021). In addition, there are differences in the molecular structures and biological characteristics of these different PD-1 inhibitors (Sher et al., 2008; Zappa and Mousa, 2016). Therefore, choosing from these approved PD-1 inhibitors remains a challenge in clinical practice. Although there are numerous clinical trials of these PD-1 inhibitors (Gandhi et al., 2018; Paz-Ares et al., 2018; Yang et al., 2020; Wang et al., 2021; Zhou et al., 2021; Ren et al., 2022; Wang et al., 2023), direct comparisons between them are rare (Fessas et al., 2017; Wu et al., 2022).

To date, only one phase II randomized controlled clinical trial (CTONG 1901) has directly compared the efficacy of two PD-1 inhibitors (sintilimab and pembrolizumab) as first-line treatments for advanced NSCLC (Liu et al., 2022). The primary endpoint of this previous study was objective response rate (ORR), and the results obtained showed similar efficacy for sintilimab and pembrolizumab. In several retrospective studies, the efficacies of PD-1 inhibitors as first-line treatment for advanced NSCLC have been compared. The results of our previous retrospective study indicated no substantial differences in progression-free survival (PFS) between pembrolizumab and nivolumab in patients with recurrent or advanced NSCLC (Cui et al., 2020; Liu et al., 2022). Further, a retrospective cohort study showed no significant difference in ORR and PFS between sintilimab and pembrolizumab as first-line treatment for advanced NSCLC (Wu et al., 2022). Another real-world study conducted by members of our research team demonstrated that sintilimab plus chemotherapy and pembrolizumab plus chemotherapy as first-line treatments for advanced squamous lung cancer show similar PFS and ORR (Yang et al., 2023).

Lung cancer is the second most common cancer type and the leading cause of cancer-related deaths worldwide (Sung et al., 2021). Non-small cell lung cancer accounts for 85% of all lung cancer cases, and approximately 70% of patients with NSCLC are diagnosed when the disease is at advanced stages (Chen et al., 2022; Zhang et al., 2023). Furthermore, choosing the optimal PD-1 therapy for patients with advanced NSCLC is crucial. Therefore, building upon previous research, we conducted a multicenter, head-to-head real-world retrospective study to investigate differences in efficacy among the six PD-1 inhibitors that are frequently used as first-line treatment for advanced NSCLC in clinical practice. This study has several advantages. First, it has a substantially larger sample size, representing a 5-13-fold larger sample size relative to the sample sizes adopted in previous studies. Second, it comprehensively covers the six commonly used PD-1 inhibitors in China. Finally, our analysis included additional overall survival (OS) and PFS data to supplement the previously lacking long-term survival benefit data.

## 2 Materials and methods

### 2.1 Participants

This study included data from patients with advanced NSCLC who received first-line PD-1 inhibitor treatment (monotherapy or in combination with chemotherapy) at the First, Third, and Fifth Medical Centers of the Chinese PLA General Hospital between June 2015 and April 2023. The enrolled patients were all aged >18 years, pathologically or cytologically confirmed to have NSCLC, and staged according to the International Association for the Study of Lung Cancer (8th edition) as having stage IIIB/IIIC (locally advanced) or stage IV (distant metastasis) NSCLC. Based on the Response Evaluation Criteria in Solid Tumors (RECIST, version 1.1), the patients were required to have at least one measurable lesion for assessment, an Eastern Cooperative Oncology Group performance status score between 0 and 2, receipt of at least one cycle of first-line PD-1 inhibitor treatment, and completion of at least one follow-up assessment. The main exclusion criteria included a lack of pathological or cytological diagnosis and NSCLC combined with other malignant tumors. Given that the study was a real-world retrospective study, individual consent was waived. The procedures for data collection were in accordance with the Declaration of Helsinki and approved by the Institutional Review Board of PLA General Hospital, Beijing, China (approval number: S2018-092-01).

### 2.2 Treatment regimen

The patients whose data were retrospectively collected in this study had received the following treatments in the past: intravenous injections of pembrolizumab, sintilimab, tislelizumab, or camrelizumab (200 mg) every 3 weeks, or intravenous injections of nivolumab or toripalimab (3 mg/kg) every 2 weeks. For patients with advanced non-squamous NSCLC, chemotherapy regimens included the intravenous administration of pemetrexed (500 mg/m^2^) as monotherapy or combination therapy comprising pemetrexed (500 mg/m^2^) with platinum-based agents (including carboplatin AUC 5 mg/mL/min; cisplatin 75 mg/m^2^, *etc.*). Further, for patients with advanced squamous lung cancer, chemotherapy regimens included paclitaxel (175 mg/m^2^), albumin-bound paclitaxel (100 mg/m^2^), gemcitabine (1250 mg/m^2^), or docetaxel (75 mg/m^2^), with or without platinum-based agents. The specific treatment plan was determined by the treating clinician based on individual circumstances.

### 2.3 Evaluation of treatment efficiency

The evaluation of tumor treatment response in the patients was based on the RECIST 1.1 criteria and assessed using magnetic resonance imaging of the brain and computed tomography scans of the chest and abdomen. The assessment criteria for treatment response included stable disease (SD), partial response (PR), complete response (CR), and progressive disease (PD). The primary endpoints for this study were PFS, defined as the time interval from the initiation of first-line treatment to disease progression or death, and OS, defined as the time interval from the initiation of first-line treatment to death from any cause. The secondary endpoints were the ORR, defined as the sum of PR and CR, and disease control rate (DCR), defined as the sum of SD, PR, and CR rates. We retrospectively collected the baseline characteristics and clinical data of the patients, including sex, age, smoking status, genetic mutations, histological type, tumor metastasis, TNM stage, PD-L1 expression, treatment cycles, progression, and death dates. At least one follow-up assessment was conducted for each patient, and the last follow-up was performed on 11 October 2023. In cases of dispute in the data collection and evaluation process, the final judgment was made by consulting designated experts (ZL and YH).

### 2.4 Statistical analysis

The chi-squared or Fisher’s exact test (for categorical variables) was used to compare the baseline characteristics and efficacy data among the different treatment groups. Kaplan–Meier survival curves and log-rank tests were used to evaluate PFS and OS for each treatment group using the Cox regression model to assess hazard ratios and 95% confidence intervals (CI). Statistical significance was set at a *p*-value <0.05. Statistical analyses were conducted using GraphPad Prism 7.0 and SPSS 25.0.

## 3 Results

### 3.1 Patient characteristics

We retrospectively collected data for 913 patients with advanced NSCLC who received first-line PD-1 inhibitor treatment (monotherapy or combined with chemotherapy). Among them, 123 (13.4%), 421 (46.1%), 239 (26.2%), 64 (7.0%), 39 (4.2%), and 27 (3.0%) received nivolumab, pembrolizumab, sintilimab, tislelizumab, toripalimab, and camrelizumab, respectively, as shown in Table 1. The baseline characteristics of the six groups were well-balanced. Further, the median ages (ranges) of patients in the nivolumab, pembrolizumab, sintilimab, tislelizumab, toripalimab, and camrelizumab groups were 64 (31–87 years), 64 (20–87 years), 63 (3–82 years), 64 (22–79 years), 62 (20–82 years), and 65 years (34–79 years), respectively. In all six groups, the proportion of males was higher than that of females. Furthermore, the nivolumab, pembrolizumab, sintilimab, tislelizumab, toripalimab, and camrelizumab groups had 71, 295, 163, 46, 30, and 22 smokers, respectively. Our analysis also indicated that most patients in the six groups had stage IV NSCLC, which accounted for 65.9%, 78.1%, 72.8%, 70.3%, 71.8%, and 70.4% of the patients in the nivolumab, pembrolizumab, sintilimab, tislelizumab, toripalimab, and camrelizumab groups, respectively. The percentages of patients with stage IIIB/IIIC disease in these six groups were 34.1%, 21.9%, 27.2%, 29.7%, 28.2%, and 29.6% in the six groups, respectively. In the nivolumab, pembrolizumab, sintilimab, tislelizumab, toripalimab, and camrelizumab groups, the proportions of patients with squamous lung cancer were 42.3%, 49.9%, 45.6%, 56.3%, 28.2%, and 51.9%, respectively, while the proportions of patients with non-squamous NSCLC were 57.7%, 50.1%, 54.4%, 43.7%, 71.8%, and 48.1%, respectively. The proportions of patients with brain metastasis, liver metastasis, bone metastasis, and adrenal gland metastasis also varied among the groups. In terms of baseline PD-L1 testing, a total of 494 patients were tested. In the nivolumab group, 16, 23, and 13 patients showed negative PD-L1 expression (PD-L1<1%), low PD-L1 expression (1%≤PD-L1<50%), and high PD-L1 expression (PD-L1≥50%). In the pembrolizumab group, 60, 107, and 85 patients showed negative, low, and high PD-L1 expression, respectively. In the sintilimab group, 41, 61, and 19 patients showed negative, low, and high PD-L1 expression, respectively. Further, in the tislelizumab group, 16, 16, and 3 patients showed negative, low, and high PD-L1 expression, respectively. In the toripalimab group, 4, 10, and 6 patients showed negative, low, and high PD-L1 expression, respectively, and in the camrelizumab group, 3, 5, and 6 patients showed negative, low, and high PD-L1 expression, respectively.

**TABLE 1:** Baseline characteristics of patients.

Characteristic	Nivolumab (*n* = 123)	Pembrolizumab (*n* = 421)	Sintilimab (*n* = 239)	Tislelizumab (*n* = 64)	Toripalimab (*n* = 39)	Camrelizumab (*n* = 27)	*p*-value
Age							0.838
<65	64 (52.0%)	216 (51.3%)	132 (55.2%)	36 (56.2%)	23 (59.0%)	13 (48.1%)	
≥65	59 (48.0%)	205 (48.7%)	107 (44.8%)	28 (43.8%)	16 (41.0%)	14 (51.9%)	
Sex							0.036
Male	96 (78.0%)	361 (85.7%)	185 (77.4%)	56 (87.5%)	32 (82.1%)	25 (92.6%)	
Female	27 (22.0%)	60 (14.3%)	54 (22.6%)	8 (12.5%)	7 (17.9%)	2 (7.4%)	
Smoking history							0.053
Yes	71 (57.7%)	295 (70.1%)	163 (68.2%)	46 (71.9%)	30 (76.9%)	22 (81.5%)	
No	52 (42.3%)	126 (29.9%)	76 (31.8%)	18 (28.1%)	9 (23.1%)	5 (18.5%)	
Stage							0.116
IIIB/IIIC	42 (34.1%)	92 (21.9%)	65 (27.2%)	19 (29.7%)	11 (28.2%)	8 (29.6%)	
IV	81 (65.9%)	328 (78.1%)	174 (72.8%)	45 (70.3%)	28 (71.8%)	19 (70.4%)	
Pathological type							0.058
Squamous lung cancer	52 (42.3%)	210 (49.9%)	109 (45.6%)	36 (56.3%)	11 (28.2%)	14 (51.9%)	
Non-squamous NSCLC	71 (57.7%)	211 (50.1%)	130 (54.4%)	28 (43.7%)	28 (71.8%)	13 (48.1%)	
Metastasis sites							
Brain	13 (10.6%)	57 (13.5%)	30 (12.6%)	3 (4.7%)	10 (25.6%)	3 (11.1%)	0.072
Liver	17 (13.8%)	41 (9.7%)	17 (7.1%)	3 (4.7%)	3 (7.7%)	1 (3.7%)	0.255
Bone	40 (32.5%)	120 (28.5%)	69 (28.9%)	21 (32.8%)	11 (28.2%)	10 (37.0%)	0.869
Adrenal gland	11 (8.9%)	40 (9.5%)	20 (8.4%)	6 (9.4%)	4 (10.3%)	0 (0.0%)	0.684
PD-L1							0.001
<1%	16 (13.0%)	60 (14.3%)	41 (17.2%)	16 (25.0%)	4 (10.3%)	3 (11.1%)	
1%–49%	23 (18.7%)	107 (25.4%)	61 (25.5%)	16 (25.0%)	10 (25.6%)	5 (18.5%)	
≥50%	13 (10.6%)	85 (20.2%)	19 (7.9%)	3 (4.7%)	6 (15.4%)	6 (22.2%)	
Not examined	71 (57.7%)	169 (40.1%)	118 (49.4%)	29 (45.3%)	19 (48.7%)	13 (48.2%)	
Treatment strategy							0.224
Combination	106 (86.2%)	335 (79.6%)	189 (79.1%)	45 (70.3%)	32 (82.1%)	22 (81.5%)	
Monotherapy	17 (24.8%)	86 (20.4%)	50 (20.9%)	19 (29.7%)	7 (17.9%)	5 (18.5%)	

NSCLC, non-small cell lung cancer.

Regarding treatment regimens, most patients in the six groups received combination chemotherapy. Particularly, in the nivolumab group, 106 patients received nivolumab plus chemotherapy, while 17 received nivolumab monotherapy. In the pembrolizumab group, 335 patients received pembrolizumab plus chemotherapy, while 86 patients received pembrolizumab monotherapy. In the sintilimab group, 189 patients received sintilimab plus chemotherapy, while 50 patients received sintilimab monotherapy. In the tislelizumab group, 45 patients received tislelizumab plus chemotherapy, while 19 patients received tislelizumab monotherapy. Additionally, in the toripalimab group, 32 patients received toripalimab plus chemotherapy, while 7 patients received toripalimab monotherapy and in the camrelizumab group, 22 patients received camrelizumab plus chemotherapy, while 5 patients received camrelizumab monotherapy (Table 1).

### 3.2 Efficacy

In patients with CR, the nivolumab group had 0 (0.0%), the pembrolizumab group had 6 (1.4%), the sintilimab group had 2 (0.8%), the tislelizumab group had 1 (1.6%), the toripalimab group had 0 (0.0%), and the camrelizumab group had 0 (0.0%), with the highest CR rate in the pembrolizumab group. For patients with PR, the nivolumab group had 53 (43.1%), the pembrolizumab group had 229 (54.4%), the sintilimab group had 133 (55.7%), the tislelizumab group had 39 (60.9%), the toripalimab group had 25 (64.1%), and the camrelizumab group had 19 (70.4%), with the highest PR rate in the camrelizumab group. In patients with SD, the nivolumab group had 62 (50.4%), the pembrolizumab group had 154 (36.6%), the sintilimab group had 93 (38.9%), the tislelizumab group had 20 (31.3%), the toripalimab group had 11 (28.2%), and the camrelizumab group had 8 (29.6%), with the highest SD rate in the nivolumab group. Regarding patients with PD, the nivolumab group had 8 (6.5%), the pembrolizumab group had 32 (7.6%), the sintilimab group had 11 (4.6%), the tislelizumab group had 4 (6.2%), the toripalimab group had 3 (7.7%), and the camrelizumab group had 0 (0.0%), with the highest PD rate in the toripalimab group. The camrelizumab group had the highest ORR (70.4%) and DCR (100.0%). The six groups showed significant differences in ORR (*p* < 0.05) but showed no significant difference in DCR (*p* > 0.05) (Table 2).

**TABLE 2:** Short-term efficacy of PD-1 inhibitors as first-line treatment for advanced NSCLC.

Efficacy measure	Nivolumab (*n* = 123)	Pembrolizumab (*n* = 421)	Sintilimab (*n* = 239)	Tislelizumab (*n* = 64)	Toripalimab (*n* = 39)	Camrelizumab (*n* = 27)	*p*-value
CR	0 (0.0%)	6 (1.4%)	2 (0.8%)	1 (1.6%)	0 (0.0%)	0 (0.0%)	
PR	53 (43.1%)	229 (54.4%)	133 (55.7%)	39 (60.9%)	25 (64.1%)	19 (70.4%)	
SD	62 (50.4%)	154 (36.6%)	93 (38.9%)	20 (31.3%)	11 (28.2%)	8 (29.6%)	
PD	8 (6.5%)	32 (7.6%)	11 (4.6%)	4 (6.2%)	3 (7.7%)	0 (0.0%)	
ORR	43.1%	55.8%	56.5%	62.5%	64.1%	70.4%	0.027
DCR	93.5%	92.4%	95.4%	93.8%	92.3%	100.0%	0.540

Abbreviations: CR, complete response; PR, partial response; SD, stable disease; PD, progressive disease; ORR, objective response rate; DCR, disease control rate; NSCLC, non-small cell lung cancer.

### 3.3 Long-term survival

**FIGURE 1:**
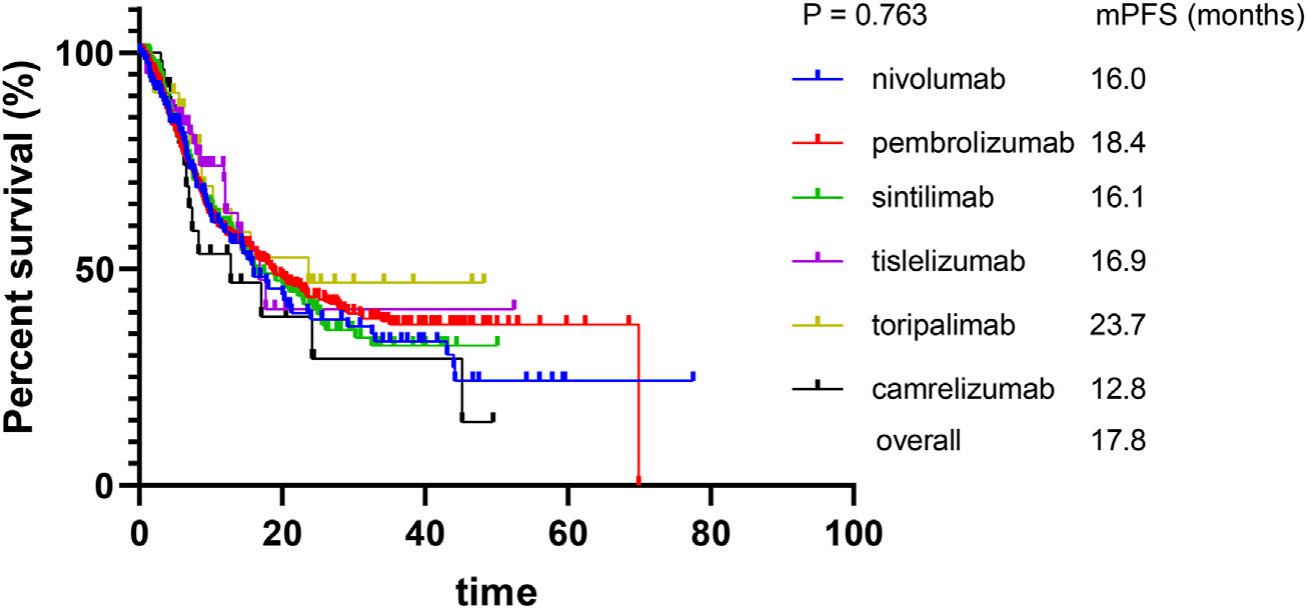
Kaplan–Meier curves for progression-free survival.

**FIGURE 2:**
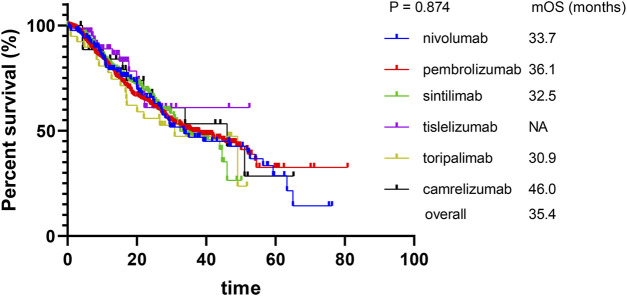
Kaplan–Meier curves for overall survival.

The median PFS and OS for the nivolumab, pembrolizumab sintilimab, toripalimab, and camrelizumab groups were 16.0 and 33.7, 18.4 and 36.1, 16.1 and 32.5, 23.7 and 30.9, and 12.8 and 46.0 months, respectively. For the tislelizumab group, the median PFS was 16.9 months, while OS was not reached. As shown in Figure 1, pairwise comparisons among the six PD-1 inhibitor groups showed no significant differences in PFS (*p* > 0.05), while the head-to-head hazard ratios are shown in Table 3. Similarly, as shown in Figure 2, pairwise comparisons among the six PD-1 inhibitor groups also revealed no significant differences in OS (*p* > 0.05), while the head-to-head hazard ratios are shown in Table 4. We further compiled the key clinical trials of the six approved PD-1 inhibitors (Table 5).

**TABLE 3:** Hazard ratios of PFS for PD-1 Inhibitors.

PD-1 inhibitors	Hazard ratio (95% CI)	PD-1 inhibitors	Hazard ratio (95% CI)	PD-1 inhibitors	Hazard ratio (95% CI)
nivolumab vs. pembrolizumab	1.105 (0.825–1.481)	pembrolizumab vs. sintilimab	0.957 (0.760–1.211)	sintilimab vs. toripalimab	1.101 (0.759–2.230)
nivolumab vs. sintilimab	1.038 (0.758–1.420)	pembrolizumab vs. tislelizumab	1.142 (0.745–1.749)	sintilimab vs. camrelizumab	0.819 (0.448–1.495)
nivolumab vs. tislelizumab	1.174 (0.728–1.891)	pembrolizumab vs. toripalimab	1.249 (0.737–2.119)	tislelizumab vs. toripalimab	1.107 (0.550–2.231)
nivolumab vs. toripalimab	1.381 (0.790–2.414)	pembrolizumab vs. camrelizumab	0.768 (0.418–1.412)	tislelizumab vs. camrelizumab	0.718 (0.354–1.458)
nivolumab vs. camrelizumab	0.828 (0.460–1.564)	sintilimab vs. tislelizumab	1.136 (0.726–1.778)	toripalimab vs. camrelizumab	0.605 (0.277–1.321)

CI, confidence interval; PFS, progression-free survival.

**TABLE 4:** Hazard ratios of OS for PD-1 Inhibitors.

PD-1 inhibitors	Hazard ratio (95% CI)	PD-1 inhibitors	Hazard ratio (95% CI)	PD-1 inhibitors	Hazard ratio (95% CI)
nivolumab vs. pembrolizumab	1.009 (0.745–1.368)	pembrolizumab vs. sintilimab	1.032 (0.795–1.340)	sintilimab vs. toripalimab	0.897 (0.529–1.522)
nivolumab vs. sintilimab	0.977 (0.695–1.374)	pembrolizumab vs. tislelizumab	1.440 (0.873–2.378)	sintilimab vs. camrelizumab	1.228 (0.685–2.200)
nivolumab vs. tislelizumab	1.287 (0.721–2.296)	pembrolizumab vs. toripalimab	0.856 (0.517–1.448)	tislelizumab vs. toripalimab	0.619 (0.303–1.268)
nivolumab vs. toripalimab	0.825 (0.489–1.485)	pembrolizumab vs. camrelizumab	1.070 (0.592–1.934)	tislelizumab vs. camrelizumab	0.831 (0.364–1.897)
nivolumab vs. camrelizumab	1.089 (0.581–2.044)	sintilimab vs. tislelizumab	1.305 (0.752–2.264)	toripalimab vs. camrelizumab	1.257 (0.603–2.620)

CI, confidence interval; OS, overall survival.

**TABLE 5:** Key clinical trials of the six approved PD-1 inhibitors.

PD-1 inhibitors	Major clinical trials	Combination drugs	Patients	Sample size	ORR (%)	DCR	Median PFS (months)	Median OS (months)
Nivolumab	CheckMate 227 Hellmann et al. (2019)	Ipilimumab	NSCLC	1739	35.9	NA	5.1	17.1 (PD-L1≥1%) 17.2 (PD-L1<1%)
Pembrolizumab	KEYNOTE-189 Gandhi et al. (2018)	Pemetrexed and platinum	Non-squamous NSCLC	616	47.6	84.6%	8.8	NA
	KEYNOTE-407 Paz-Ares et al. (2018)	Carboplatin and paclitaxel/nab-paclitaxel	Squamous lung cancer	559	57.9	NA	6.4	15.9
Sintilimab	ORIENT-11 Yang et al. (2020)	Pemetrexed and cisplatin/carboplatin	Non-squamous NSCLC	397	51.9	86.8%	8.9	NA
	ORIENT-12 Zhou et al. (2021)	Gemcitabine and cisplatin	Squamous lung cancer	543	44.7	NA	5.5	NA
Tislelizumab	A Phase 3 Randomized Clinical Trial Wang et al. (2021)	Paclitaxel/nab-paclitaxel and carboplatin	Squamous lung cancer	360	72.5%/74.8	88%/91%	7.6	NA
Toripalimab	CHOICE-01 Wang et al. (2023)	Squamous NSCLC: nab-paclitaxel and carboplatin Non-squamous NSCLC: pemetrexed and cisplatin/carboplatin	NSCLC	465	65.7	NA	8.4	NA
Camrelizumab	CameL-Sq Ren et al. (2022)	paclitaxel and carboplatin	Squamous lung cancer	390	64.8	NA	8.5	NA

DCR, disease control rate; ORR, objective response rate; OS, overall survival; PFS, progression-free survival; NSCLC, non-small cell lung cancer.

## 4 Discussion

An increasing number of PD-1 inhibitors have been approved as first-line treatment of advanced NSCLC, and in clinical trials, these drugs have demonstrated superior efficacy relative to standard chemotherapy, providing clinicians with more treatment options. However, the comparative efficacies of these different PD-1 inhibitors remain uncertain. Further, authoritative guidelines are yet to recommend a specific PD-1 inhibitor as more suitable for patients with advanced NSCLC. Clinical trials have strict eligibility criteria, and most often, the enrolled patients do not fully represent the heterogeneity of real-world patients. Unlike randomized controlled trials, real-world patients receiving PD-1 inhibitors may include older patients, those with multiple comorbidities, and those with other underlying conditions (Wu et al., 2022), thus necessitating the further validation of the efficacies of different PD-1 inhibitors in real-world settings. Our study directly compared the efficacies of the six major PD-1 inhibitors as first-line treatment for advanced NSCLC for the first time. Our results demonstrated that nivolumab, pembrolizumab, sintilimab, tislelizumab, toripalimab, and camrelizumab have comparable efficacy as first-line treatment options for advanced NSCLC.

We also noted that pembrolizumab, sintilimab, and nivolumab are the most used PD-1 inhibitors in real-world clinical practice, with their use accounting for 46.1% (421/913), 26.1% (239/913), and 13.4% (123/913) of the clinical population, respectively. Additionally, tislelizumab, toripalimab, and camrelizumab have also been used to varying degrees in clinical practice. Our study results indicated that for short-term efficacy, there are differences in ORR among the different PD-1 inhibitors, but no significant differences were observed with respect to DCR. PD-L1 expression levels have been demonstrated to be positively correlated with clinical benefits for patients with NSCLC receiving PD-1 inhibitor therapy (Wu and Lu, 2020; Powell et al., 2021). The difference in ORR may be due to imbalances in sample size and differences in PD-L1 expression across groups. In our study, the camrelizumab group had the highest proportion of patients with high PD-L1 expression (22.2%), which resulted in the highest ORR and DCR values (70.4% and 100.0%, respectively).

Survival benefits are the gold standard for clinical treatment evaluation, and in this study, we presented median PFS and OS data for the six PD-1 inhibitors based on real-world data. The study results also showed no statistically significant differences in PFS and OS among the six groups of PD-1 inhibitors. However, we observed higher PFS and OS than was reported in previously published clinical trials (Table 3). A possible reason for this discrepancy is the larger sample size and higher PD-L1 expression levels in this study, which possibly affected long-term survival outcomes (Maggie Liu et al., 2023). For example, among the patients who underwent PD-L1 testing before first-line PD-1 treatment, 76.2% (192/252) in the pembrolizumab group and 69.2% (36/52) in the nivolumab group showed PD-L1 expression ≥1%, higher than the proportions reported in previous clinical trials (Paz-Ares et al., 2018; Yang et al., 2020). Previous studies have also shown that in NSCLC, PD-L1 expression tends to be moderately to highly expressed (Wu and Lu, 2020; Powell et al., 2021). Furthermore, this study included a large proportion of patients with locally advanced NSCLC. Phase III clinical trials have shown that the addition of durvalumab after concurrent chemoradiotherapy leads to a median PFS of 17.2 months and a median OS of 47.5 months in this group of patients with unresectable stage III NSCLC (Zhou et al., 2021). Therefore, the use of PD-1 inhibitors in real-world settings may also result in longer survival benefits for these patients. Finally, treatment strategies in real-world clinical practice may differ from those in clinical trials. Unlike the combination chemotherapy regimens with the respective PD-1 inhibitors listed in Table 3, treatment regimens in the real-world setting are more diverse. For example, in the sintilimab group in this study, the combination chemotherapy regimen included nab-paclitaxel. In the pembrolizumab group, patients also received various feasible combination regimens, including paclitaxel/nab-paclitaxel with carboplatin/cisplatin/lobaplatin; gemcitabine with platinum agents; and docetaxel with platinum agents. These variations in combination therapies may have contributed to the differences in patient survival between our study and previously reported clinical trials. However, our study findings are consistent with those of a previous single-center retrospective study in which the efficacies of pembrolizumab and sintilimab as first-line treatments for advanced squamous lung cancer were compared (Yang et al., 2023). In this previous study, the observed median PFS was 22.2 months in the sintilimab group and 16.5 months in the pembrolizumab group, similar to the results obtained in the present study.

The effect of the different mechanisms of action as well as structural differences among different PD-1 inhibitors on their therapeutic efficacies is an area of research interest. Although pembrolizumab, sintilimab, and other PD-1 inhibitors are all IgG4 antibodies, their binding sites are different. For example, pembrolizumab primarily binds to the C'D loop on PD-1 on human T cells, which confers high PD-1 specificity and affinity, while the main binding site of sintilimab is the FG loop (Yang et al., 2023). Some studies have suggested that the fully humanized structure of sintilimab helps to reduce immunogenicity and the risk of infusion reactions (Wang et al., 2021; Ren et al., 2022; Wang et al., 2023). In summary, while different immunotherapy drugs have demonstrated efficacy, the influence of differences in structure and biological characteristics on their efficacy remains unclear.

Our study included a total of 913 patients and showed that although there were slight numerical differences in PFS and OS among the six PD-1 inhibitors, the differences were not statistically significant, suggesting that the choice of PD-1 inhibitors may be based on patient and physician preferences. Regardless, our study had some limitations. First, our results are possibly associated with information and selection biases as it was a retrospective analysis conducted at three centers. Second, not all patients underwent PD-L1 expression testing before receiving first-line PD-1 inhibitor treatment. Third, our study only included Chinese patients. Thus, the results may not be apply to other populations. Fourth, this study mainly focuses on the efficacy of different PD-1 inhibitors and does not compare the immune-related adverse events associated with different PD-1 inhibitors. Immune-related adverse events also hold significant value in drug selection.

In in this real-world retrospective study, we demonstrated that in clinical practice, nivolumab, pembrolizumab, sintilimab, tislelizumab, toripalimab, and camrelizumab show similar efficacy as first-line treatment options for advanced NSCLC, with comparable long-term survival outcomes.

## Data Availability

The original contributions presented in the study are included in the article/Supplementary material, further inquiries can be directed to the corresponding authors.
